# Clinical actionability in gliomas revealed by real-world next-generation sequencing: a multicentric study

**DOI:** 10.1038/s41698-025-01247-3

**Published:** 2026-01-06

**Authors:** Oriol Mirallas, Fiorella Ruiz-Pace, Gabriel Velilla, Diego Gómez-Puerto, Teresa Gorria, Jesus Yaringaño, Álvaro Martínez-Monino, Daniel López-Valbuena, Maria Angeles Vaz, Ainhoa Hernandez, Elena Martínez-Saez, Maria Aguado Sorolla, Maria Castro-Henriques, Julia Lostes Bardaji, Antonio Di Muzio, Oscar Gallego, María Martínez-García, Marta Domenech, Joan Carles, Carmen Balaña, Estela Pineda, Rodrigo Dienstmann, Juan Manuel Sepúlveda, Maria Vieito

**Affiliations:** 1 Medical Oncology Department, Vall d’Hebron University Hospital and Vall d’Hebron Institute of Oncology (VHIO), Barcelona, Spain; 2 Oncology Data Science (ODysSey) Group, Vall d’Hebron Institute of Oncology, Barcelona, Spain; 3 Neuro-Oncology Unit, Instituto de Investigación 12 de Octubre, Madrid, Spain; 4 Medical Oncology Department, IDIBAPS, Hospital Clinic, Barcelona, Spain; 5 Medical Oncology Department, Hospital del Mar, Barcelona, Spain; 6 Medical Oncology Department, Hospital Ramón y Cajal, Irycis, Madrid, Spain; 7 Medical Oncology Department, Institut Catala d’Oncologia (ICO) Badalona, Badalona Applied Research Group in Oncology (B-ARGO Group), Institut Investigació Germans Trias i Pujol (IGTP), Badalona, Spain; 8 Pathology Department, Vall d’Hebron University Hospital, Barcelona, Spain; 9 Medical Oncology, Hospital de de la Santa Creu i Sant Pau, Barcelona, Spain; 10 Humanitas University, Milan, Italy; 11 University of Vic – Central University of Catalonia, Barcelona, Spain

**Keywords:** CNS cancer, Tumour biomarkers, Tumour heterogeneity, Molecular medicine, Drug development

## Abstract

Treatment options for patients with gliomas remain limited, and prognosis is generally poor. While next-generation sequencing (NGS) is increasingly used to stratify glioma patients and guide therapy, its implementation in routine clinical practice remains variable. We conducted a multicenter retrospective study across seven Spanish hospitals to evaluate the clinical utility of NGS in glioma management, focusing on its impact on diagnosis and treatment selection based on the ESMO Scale for Clinical Actionability of Molecular Targets (ESCAT). A total of 541 glioma patients diagnosed between 2018 and 2022 were included; 76% had glioblastomas and 24% other glioma subtypes. Among glioblastoma patients, 9% harbored ESCAT tier 1/2 alterations and 74% tier 3/4. Molecularly matched therapy was administered in 10.2% of glioblastoma cases. Objective responses were observed in 17.6% of glioblastoma and 33% of non-glioblastoma patients with ESCAT tier 1/2 alterations. Patients with tier 1/2 alterations experienced significantly longer progression-free survival compared to those with tier 3/4. These findings support genomic profiling of gliomas in research centers to expand therapeutic options in molecularly guided clinical trials.

## Introduction

Central nervous system (CNS) tumors comprise a diverse group of over 40 entities. Even if considered as a single entity, they would meet the criteria for rare tumors, with an estimated incidence of 308,102 new cases and 251,329 deaths worldwide in 2020^1^. The World Health Organization (WHO) classification of malignant CNS tumors used to rely on histological findings, such as morphology and immunohistochemical features. However, new molecular biomarkers have improved tumor classification, leading to more precise diagnoses^2–4^. The WHO 2021 classification integrated molecular data as key elements to guide diagnosis and treatment.

Glioblastoma, the most common malignant tumor in adults, accounts for 49% of malignant brain tumors. Treatment remains challenging and varies by tumor type and location. Surgery is the first-line treatment, with complete resection as the primary goal^5^. Adjuvant treatment with radiation and temozolomide-based chemotherapy remains the standard, based on the phase III trial by Stupp et al.^6^. Targeted therapies have emerged for specific brain tumors, often developed in tumor-agnostic clinical trials. For example, the tissue-agnostic approval of dabrafenib and trametinib for BRAFV600E-mutated gliomas followed the positive results of the ROAR basket trial^7,8^. More recently, the INDIGO phase III clinical trial with vorasidenib demonstrated efficacy in *IDH1/2-*mutant type 2 gliomas surgery^9,10^.

The ESMO Scale of Clinical Actionability for Molecular Targets (ESCAT)^11^ prioritizes genomic alterations for targeted therapies. The ESMO Magnitude of Clinical Benefit Scale (MCBS) assesses the clinical benefit of treatments based on progression-free survival (PFS) and overall response rate (ORR)^12^. An initial ESCAT proposal for gliomas was presented at ESMO 2022^13^, followed by the European Association of Neuro-Oncology (EANO) guidelines on rational molecular testing in gliomas. The 2023 version of these guidelines did not support the broad use of NGS given its limited clinical actionability. However, in the updated 2025 guidelines, the recommendation was revised: NGS is now considered indispensable for diagnostic purposes, while its therapeutic utility remains limited, with only IDH mutations and BRAF V600E/fusions recognized as validated actionable alterations in specific contexts^14^. This initial assessment will evolve as more targeted treatments receive regulatory approval.

The Cancer Genome Atlas Research Network (TCGA) established the genomic and transcriptomic landscape of glioblastoma^15^. In 2010, Verhaak et al. identified four distinct molecular subtypes (proneural, neural, classical, and mesenchymal) with different clinical outcomes and treatment sensitivities^16^. Subsequent work by Brennan et al. expanded this classification using DNA methylation, miRNA expression, and protein analysis, identifying key pathways like *EGFR* and *PDGFRA*. Despite advances in glioma molecular profiling, real-world implementation remains limited due to challenges in standardizing multiparametric testing, though key mutations aid clinical distinction^17^.

While NGS is not standard-of-care for glioma diagnosis, the WHO 2021 classification requires pathologists to conduct a series of tests, which are costly and require substantial tissue. In contrast, NGS can detect multiple alterations in a single test, offering new opportunities for molecularly matched therapies. Early-phase trials with BRAF, NTRK, and IDH inhibitors have shown safety and efficacy in glioma patients^7,10,18^. Despite this, the number of glioma patients included in trials remains low, highlighting the need for increased molecular testing and real-world evidence supporting targeted therapies.

To address this gap, we analyzed a large CNS tumor cohort undergoing routine NGS to assess ESCAT’s role in therapy prioritization and the clinical impact of molecular-matched treatments.

## Results

### Patient population

**Fig. 1:**
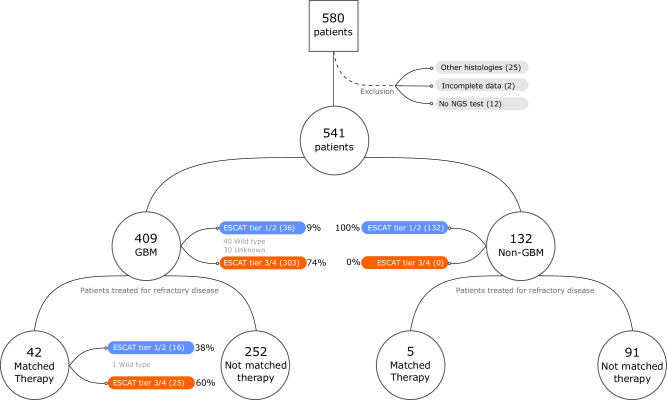
Flow chart of the study population.

From January 2018 to May 2022, 580 primary brain tumors patients were included; 541 glioma patients met eligibility criteria (Fig. 1). The median number of treatment lines was 3 (range 1–10). Median age was 51 years (range 3–84), with glioblastoma patients being older than non-glioblastoma [55 vs 36 years, *p* < 0.001). No differences were found by sex, MGMT methylation, or ECOG/Karnofsky performance status (PS) (*p* > 0.05). Over 80% of patients underwent surgery, with no significant in complete vs incomplete resection between glioblastoma vs non-glioblastoma (*p* = 0.055). After surgery, 79% of glioblastoma patients received Stupp protocol chemoradiation vs 46% of non-glioblastoma patients (Supplementary Table 1).

### ESCAT proposal for glioma

The ESCAT proposal for gliomas and MCBS is based on published efficacy data, supplemented by the real-world distribution of mutations in our cohort (Table 1). Among 409 glioblastoma cases with NGS, 83% had actionable alterations: 9% (*n* = 36) were tier 1/2 (IDH1/2, BRAF V600E, FGFR1-3 mutations/rearrangements, NTRK1-3 fusions), and 74% (*n* = 303) were tier 3/4 alterations (Table 1). Matched therapy was given to 42 glioblastoma (10.2%) and 5 non-glioblastoma (3.8%) patients. These results show that integrating NGS increases eligibility for targeted therapies.

**Table 1:** ESCAT proposal for glioma tumors according to the most recent evidence

ESCAT	Alteration	Matched therapy and ESMO MCBS	Patients with alteration	Patients tested	Prevalence	Altered patients by ESCAT	Total by group
Tier 1	BRAF - V600E mutation	Dabra-Trame: 3	7	497	1,4%	144	168
	IDH1 mutation	Vorasidenib: 3	123	535	23,0%		31,1%
	IDH2 mutation	Vorasidenib: 3	10	492	2,0%		
	NTRK (1-3) fusion	Larotrecitnib/ Entrectinib: ND	10	489	2,0%		
Tier 2	FGFR (1-3) pathogenic mutations	Erdafitinib: 3	14	482	2,9%	24	
	FGFR rearrangement (FGFR-TACC)	Futibatinib/ Pemigatinib: 3	13	473	2,7%		
Tier 3	AKT1 - E17K mutation	-	0	467	0,0%	153	303
	BRAF fusions^a^	Selumitinib: ND	8	495	1,6%		56,0%
	FGFR (1-3) amplification	Erda/Futi/Pemi: ND	3	459	0,7%		
	H3K27M mutation	-	11	455	2,4%		
	MET mutation	Vebreltinib/ Capmatinib: ND	10	469	2,1%		
	PI3K mutations (PIK3CA, PTEN)	Capmatinib: ND	149	492	30,3%		
	PTEN loss	-	19	479	4,0%		
Tier 4	ATM mutation	-	14	349	4,0%	150	
	ATRX mutation	ATR inhibitor: ND	80	459	17,4%		
	CDKN2A loss	-	127	445	28,5%		
	CDKN2B loss	-	108	393	27,5%		
	EGFR amplification	Depatuxizumab mafodotin/Dacomitinib: ND	128	482	26,6%		
	EGFR mutation	-	69	497	13,9%		
	EGFR vIII rearrangement	EGFRvIII-TCBA/Rindopepimut: ND	72	492	14,6%		
	pTERT mutation	-	205	437	46,9%		
Wild type			40	541	7,4%		
Unknown	Incomplete results		30	541	5,5%		
			Total	541			

*Dabra-Trame* dabrafenib-trametinib, *ND* no data.

^a^Pending results of FIREFLY-2.

### Uncovering molecular alterations and co-mutations in real-world data

All mutations encountered in the entire cohort are detailed in Supplementary Table 2. The most common *TERT* co-mutations in glioblastoma patients were *CDKN2A* loss (20%), EGFR amplification (20%), *PIK3CA* mutation (19%), and CDKN2B loss (19%) (Supplementary Fig. 1A). Most patients with *CDKN2A* loss also had CDKN2B loss [101 patients (25%)]. In patients with non-glioblastoma tumors, the most common co-mutation for *IDH1* was *ATRX* loss (48%), followed by *TERT* mutation (19%) and *PIK3CA* mutation (12%) (Supplementary Fig. 1B).

For the entire cohort, the most common mutations were found in *TERT* (47%), *TP53* (33%), *PI3KCA* (30%), and *CDKN2A*/*B* (28%). In the GBM cohort exclusively, the most common mutations were found in *TERT* (54%), *CDKN2A* (37%), *PI3KCA* (36%), *CDKN2B* and *EGFR* amplified (35% each, Supplementary Fig. 2A). The non-GBM cohort’s most common mutations were *IDH1* (93%), *TP53* (61%), *ATRX* (58%), and *TERT* (26%, Supplementary Fig. 2B).

### OS in our real-world data population

**Fig. 2:**
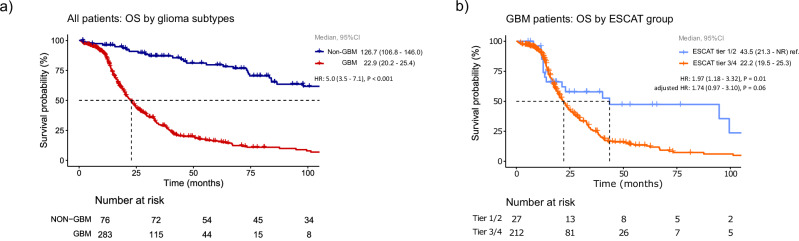
Overall survival of patients with glioma. **a** Overall survival for glioblastoma (GBM) vs non-glioblastoma (non-GBM). **b** Overall survival for glioblastoma patients harboring ESCAT tier 1/2 vs tier 3/4.

Survival was significantly worse in glioblastoma than in non-glioblastoma patients [22.9 months (95% CI 20.2-25.4) versus 126.7 months (95% CI 106.8–146; HR = 5.0 95% CI 3.5–7.1, *p* < 0.001)] (Fig. 2a). In the glioblastoma cohort, OS was shorter among patients with ESCAT tier 3/4 alterations (median 22.2 months, 95% CI 19.5–25.3) compared with those harboring tier 1/2 alterations (43.5 months, 95% CI 21.3–NR; HR = 1.97 95% CI 1.18–3.32, *p* = 0.01). After adjustment for confounders in the multivariable analysis, a trend towards worse OS persisted for ESCAT 3/4 vs 1/2 [HR = 1.74, 95% CI 0.97–3.10; *p* = 0.062, Fig. 2b].

### Clinical actionability of targeted therapy in glioma patients

**Fig. 3:**
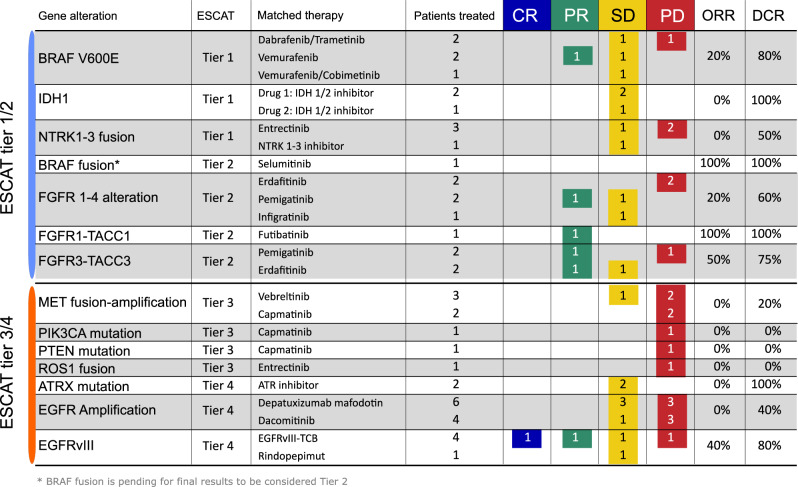
Objective response rate and disease control rate of all targeted therapy administered per molecular alteration and tier. *Legend: Targeted treatments are ordered per tier and number of patients treated. BRAF fusion is considered as tier 3 pending results of FIREFLY-2.

Overall, the ORR for targeted therapies was 11%. The ORR in ESCAT tier 1/2 patients was 26% (DCR 80%), compared to 8% in tier 3/4 (DCR 34%) population, which exhibited a DCR of 34%. *FGFR 1-3* fusions/rearrangements treated with covalent FGFR inhibitor (futibatinib, pemigatinib, erdafitinib) achieved 60% ORR and 80% DCR, whereas *FGFR 1-4* mutations had 20% ORR and 60% DCR. *BRAF V600E-*mutant patients receiving BRAF/MEK inhibitors had 20% ORR and 80% DCR, with one partial response with BRAF inhibitor alone. One BRAF fusion patient treated with selumetinib responded. IDH1 and NTRK achieved 100% and 50% DCR, respectively, but no PRs. Capmatinib failed to elicit responses in *MET* fusions or *PIK3CA*/*PTEN-*mutant cases (Fig. 3).

**Fig. 4:**
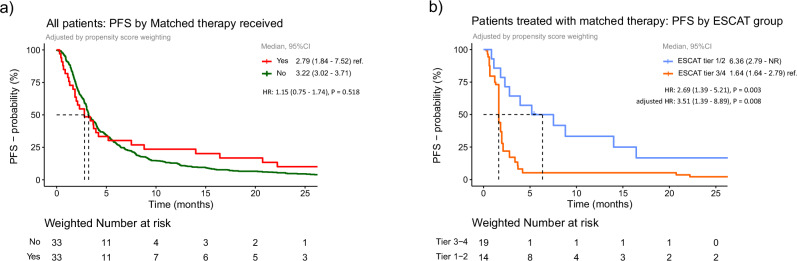
Progression-free survival curves according to molecular matched therapy (4A) and progression-free survival curves according to ESCAT (4B). *Legend: In **a**, the red line represents patients receiving molecularly matched therapy, while the green line represents those not receiving molecularly matched therapy. In **b**, the blue line corresponds to patients with ESCAT tiers 1/2 molecular alterations, and the orange line corresponds to patients with tiers 3/4 molecular alterations.

**Fig. 5:**
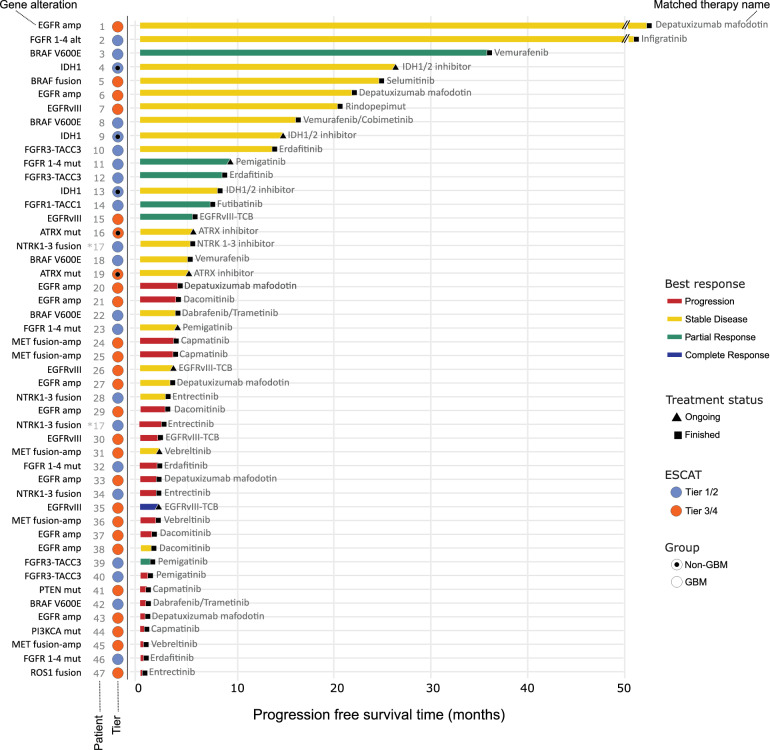
Progression-free survival according to therapy administered and matched molecular alterations, its tier according to ESCAT, and its best response rate. *Legend: Progression disease is labeled in red, stable disease in yellow, partial response in green, and complete response in blue. Black triangle stands for ongoing treatment, and black square stands for halted treatment. Blue circle stands for tiers 1/2, and orange circle stands for tiers 3/4 molecular alterations. Dot circled stand for non-GBM, and empty circle stands for GBM.

Patients with ESCAT tier 3/4 alterations experienced significantly shorter PFS compared with those with tier 1/2 alterations (1.64 vs. 6.36 months, HR = 2.69, 95% CI 1.39–5.21, *p* = 0.003), and this association remained significant after multivariate adjustment (HR = 3.51, 95% CI 1.39–8.98, *p* = 0.008; Fig. 4b). Treatment within early-phase trials did not negatively impact PFS (HR 1.15, 95% CI 0.75–1.74, *p* = 0.518) (Fig. 4a). The longest PFS durations were observed among patients harboring *FGFR 1-4* mutations*, BRAF V600E*, *IDH1 R132H*, *BRAF* fusion, *EGFR* amplification, particularly when treated with MAPK-targeted therapies (Fig. 5).

### Classification of glioblastoma patients using real-world NGS data

As an exploratory analysis, we aimed to determine whether the 409 patients in our database classified as glioblastoma could be reassigned to an adapted molecular classification based solely on their genomic features identified by NGS, without the inclusion of RNA or methylation profiling (Supplementary Fig. 3A). Of 409 glioblastoma patients, 12% exhibited proneural, 20% mesenchymal, and 68% classical molecular hallmarks, enabling classification of 72% of cases (Supplementary Fig. 3A and Supplementary Table 3). No differences in age, gender, MGMT methylation, ECOG PS, or OS were found among subtypes (Supplementary Fig. 3B).

## Discussion

Our multicenter study exploring the potential of real-world data for identifying clinically actionable targets underscores the clinical utility of NGS in glioma diagnosis and treatment decision-making. Per ESMO recommendations, NGS should be prioritized for tumors with a high likelihood of ESCAT tier 1 alterations and in hospitals with drug development programs for tiers 2–4^11^. NGS is essential not only for identifying targeted therapies in glioma but also for accurate diagnosis by updated guidelines. However, clinically actionable targets in CNS tumors remain limited despite recent advances both in diagnosis and treatment options. As recommended by EANO, we must carefully assess when and which molecular alterations to test in adult primary brain tumors^14^.

Pathological assessment should include actionable mutations such as *IDH1/2*, *CDKN2A/B* loss, *TERT* mutations, *EGFR* amplifications, and nuclear ATRX loss. IHC detects canonical *IDH* mutations and *ATRX* loss, while ISH identifies *CDKN2A/B* deletions and *EGFR* amplifications. However, NGS enhances diagnosis by detecting rare *IDH* mutations and identifying “molecular glioblastoma” in *IDH*-wildtype gliomas without microvascular proliferation or necrosis.

Moreover, our dataset showed ESCAT tier 1 in 28% and tier 2 in 6.5%, exceeding prior glioma studies (3–7%)^19,20^. Notably, 10.2% of our glioblastoma patients received molecularly matched therapy through clinical trials or as compassionate use, surpassing the 4.1% reported in the NCI-match trial across tumor types^21^. Considering the limited enrollment of glioma patients in clinical trials and the scarcity of targeted therapies, there is potential for expanding precision medicine and improving glioma patients’ outcomes.

In our real-world glioma cohort, we identified clinical and molecular factors associated with an increased likelihood of detecting relevant molecular events. Patients with ESCAT 1/2 alterations had superior PFS (6.4 vs 1.6 months) compared to those with tier 3/4 alterations (Fig. 4b). Notably, FGFR fusions/rearrangements showed the highest ORR with covalent FGFR inhibitors, consistent with NCI-MATCH trial findings^21^(Fig. 3). These insights underscore the value of understanding glioma molecular alterations in expanding treatment opportunities. ESCAT profiling may better stratify patients for novel therapies, potentially leading to superior clinical outcomes (Fig. 5).

TCGA molecular subtypes require more than NGS alone^17^, necessitating additional molecular layers. The proneural group, which includes glioblastomas with *IDH* promoter mutations or epigenetic alterations, is now classified separately by the 2021 WHO guidelines. While our data align with Verhaak’s subtypes, they do not replicate the OS reported for each group. Our study has limitations, including selection bias, a retrospective study, variable NGS timing, and platform heterogeneity, which may affect the generalizability of results. However, with over 500 glioma patients from seven hospitals across Spain, our cohort reflects real-life oncology. The inclusion of diverse treatment types strengthens the findings, and our study presents the largest clinical and genomic glioma dataset published to date. Despite NGS heterogeneity, molecular characterization allowed for accurate diagnoses and increased matched therapies, supporting its broader adoption. A prospective study will be needed to demonstrate our findings.

Our findings suggest that NGS should be integrated into glioma management to enhance diagnostic precision and identify targeted therapy opportunities in research centers with access to clinical trials. ESCAT profiling aids in selecting molecularly matched therapies, advancing personalized treatment approaches.

## Methods

### Population and study design

We conducted a retrospective analysis of clinical characteristics, NGS parameters, and matched therapies in a multicentric Spanish cohort of glioma patients treated from 2008 to 2023. The inclusion criteria were patients with glioma diagnosis, available NGS analysis and who underwent systemic treatment. The primary goal of NGS was to detect targetable mutations for clinical trial enrollment or molecularly matched targeted therapy. Data were collected via REDCap and included demographics, tumor type, surgery, systemic therapy, targeted therapy, clinical trial participation, and survival status as of May 2023. Patients were classified as glioblastoma or non-glioblastoma per WHO 2021 and further categorized using an adapted molecular classification (TCGA/Verhaak). Molecular alterations were classified by ESCAT and MCBS criteria.

### Sample collection and NGS genomic profiling

Molecular profiling was performed on tumor tissue from biopsies or surgical samples. DNA was analyzed using in-house NGS panels (21–541 genes for mutations, 26 genes for fusions using NanoString nCounter) or the FoundationOne CDx platform (324 genes, Foundation Medicine, Inc.). The panels used at each institution were as follows: Vall d’Hebron Hospital: VHIO-300 in-house panel (ISO certified) and Oncomine Focus Assay (Thermo Fisher Scientific); 12 de Octubre Hospital: FoundationOne® CDx (324 genes, DNA) and CARIS Whole Transcriptome Sequencing; Clinic Hospital: FoundationOne® CDx (324 genes, DNA) and CARIS Whole Transcriptome Sequencing; both ICO Badalona: Oncomine Precision Assay (Thermo Fisher Scientific); Sant Pau Hospital: Oncomine Focus Assay (Thermo Fisher Scientific); and Ramón y Cajal Hospital: FoundationOne® CDx (324 genes, DNA). Additional data on mismatch repair deficiency (dMMR), microsatellite instability (MSI), and tumor mutational burden (TMB) were collected.

### Statistical analysis

Descriptive statistics assessed baseline characteristics of patients, comparing groups and testing patterns. Continuous variables were expressed as median (IQR), and categorical variables as absolute values and percentages.

### Progression-free survival (PFS)

PFS, our main objective of the study, was defined as the time from the treatment initiation to disease progression or death from any cause. We compared PFS between patients receiving ESCAT tier 1/2 versus tier 3/4 therapies and between molecularly matched versus non-matched treatments. For patients who did not receive a molecularly matched therapy, treatment initiation was the first systemic therapy after completing or discontinuing the Stupp regimen, regardless of prior therapies. To control for selection bias, propensity score weighting through logistic regression was applied, including age, therapy line, tumor grade, and surgery outcome. Treatment effect was estimated using “average treatment effect for the treated”. A multivariable Cox proportional hazards (PH) model for PFS was fitted among patients who received matched therapy (GBM and non-GBM), using time from treatment initiation as the time origin. The exposure variable was ESCAT tier 3/4 versus 1/2, adjusting for age at diagnosis, sex, ECOG PS (collapsed into 0/1/2–4 to improve model stability), extent of resection, and line position of the matched therapy. Histology (GBM vs. non-GBM) was incorporated through stratification. Inverse probability of treatment weighting (IPTW) using propensity scores (*psvalue*) was applied, with robust standard errors to account for weighting. PH assumptions held (global Schoenfeld *p* = 0.79; ESCAT tier *p* = 0.28).

### Overall survival

The study’s secondary objectives were to assess the overall survival (OS) of the glioblastoma and non-glioblastoma cohorts, compare OS between ESCAT tier 1/2 and tier 3/4 groups, and evaluate OS according to molecularly adapted classification. Time-to-event endpoints were estimated using Kaplan–Meier, with comparisons via the log-rank test. To mitigate immortal bias arising from differences in patient entry times based on reported NGS test results, all estimates were adjusted using the risk set method, with the NGS test date serving as the index date. Univariate Cox PHs models were applied to calculate hazard ratios (HRs) with 95% confidence intervals (CIs). A multivariable Cox PHs model for OS was fitted in patients with GBM, with left truncation at the date of next-generation sequencing (NGS). The exposure variable was ESCAT tier 3/4 versus 1/2, adjusting for age at diagnosis, sex, and ECOG PS. Violations of the PH assumption were identified for extent of resection and total number of treatment lines in sensitivity analyses; therefore, the model was stratified by these factors. Under this specification, the PH assumption was met (global Schoenfeld *p* = 0.19; ESCAT tier *p* = 0.12).

### Objective response rate and disease control rate

ORR was defined as the proportion of patients achieving a complete response or partial response, disease control rate (DCR) included patients with stable disease (SD). *P*-values were two-sided and adjusted by the Benjamini and Hochberg (BH) method to account for multiple comparisons. Statistical analyses were conducted in R (v4.3.0).

### Ethical approval

This study was conducted in accordance with the ethical standards laid down in the 1964 Declaration of Helsinki and its later amendments. The study protocol was approved by the Institutional Review Board (IRB) of Vall d’Hebron University Hospital (PR (AG)498/2022), Catalan Institute of Oncology (ICO) Badalona (PI-23-048), Hospital del Mar (2023/10822/I), Hospital Clinic de Barcelona (HCB/2023/0720), Hospital de la Santa Creu i Sant Pau (IIBSP-MBT-2025-213), 12 de Octubre (23/091), and Hospital Ramon y Cajal (MAP-BT 341/25) in Madrid. All glioma patients with NGS performed at these centers were included. Living patients provided informed consent, while the IRB exempted deceased patients.

## Supplementary information

Supplementary_Figures_Tables.

## Data Availability

Deidentified patient data from this study can be made available to qualified investigators who provide a methodologically sound research proposal and sign a data access agreement. Please email oriolmirallas@vhio.net for information. The study protocol, statistical analysis plan, and informed consent form will also be made available upon request. Data will be shared via a secure online platform; REDcap and public repository on the VHIO website.
