# Supplementary material for: Clinical actionability in gliomas revealed by real-world next-generation sequencing: a multicentric study
Source: NPJ Precis Oncol. 2026 Jan 6;10:46. doi: 10.1038/s41698-025-01247-3 (PMC12855890; doi:10.1038/s41698-025-01247-3)
Supplement: Supplementary file 1 — Supplementary_Figures_Tables. [file 41698_2025_1247_MOESM1_ESM.pdf]

Supplementary Figures

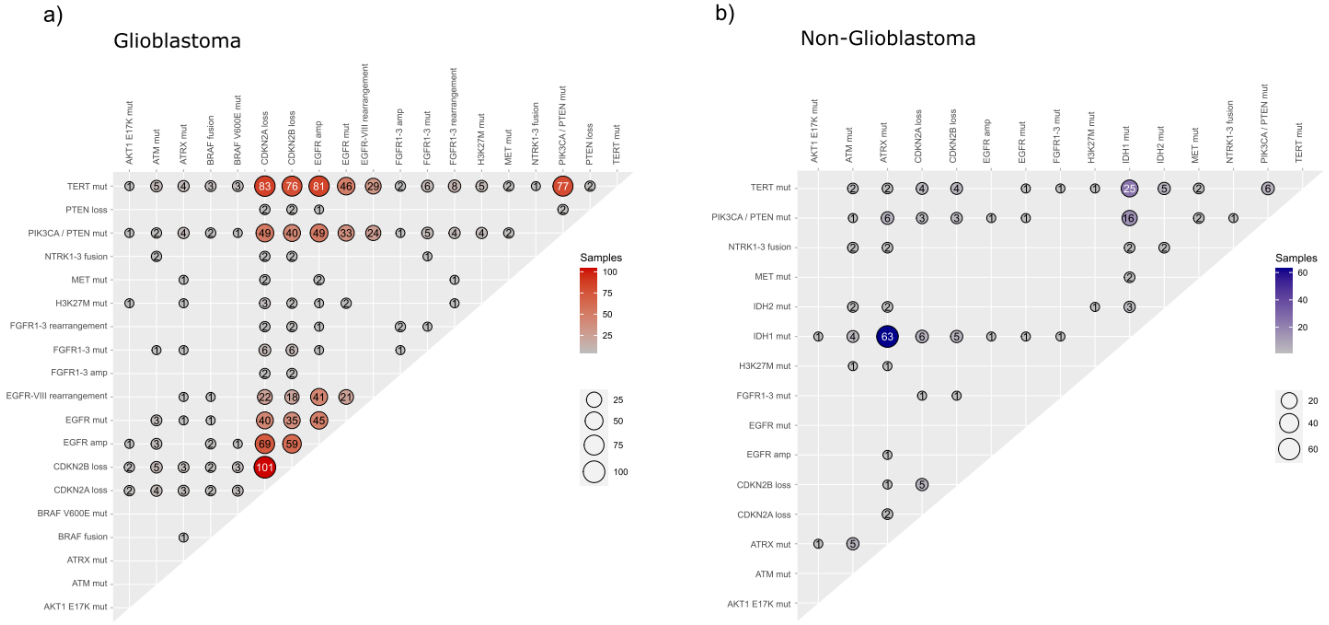

Supplementary Figure 1: Total co-mutations in GBM (A) and co-mutations in non-GBM (B).

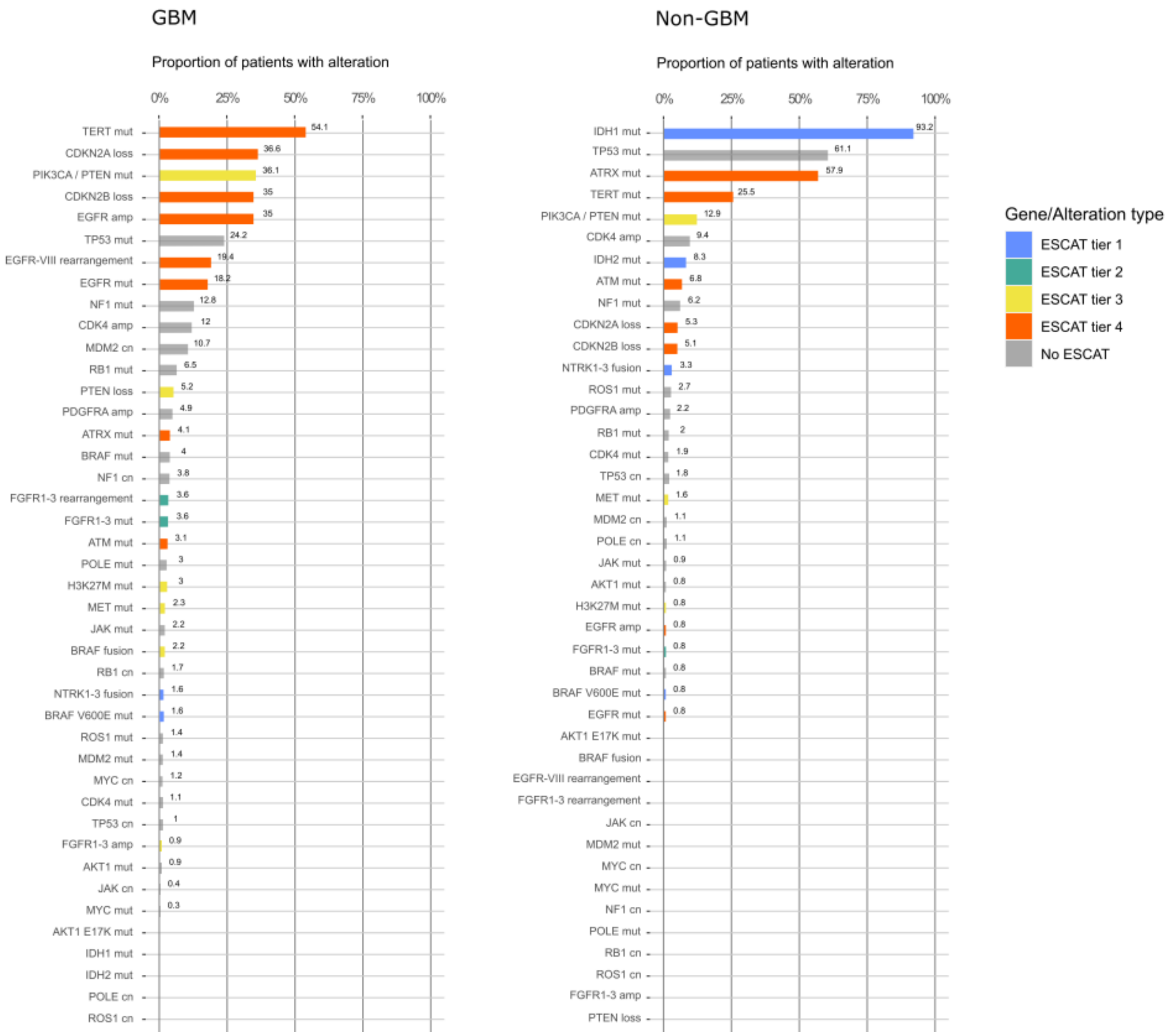

Supplementary Figure 2: Total gene alterations separated by GBM (A) and non-GBM subtypes (B).

Legend: Blue stands for Tier 1 molecular alterations, green stands for Tier 2, yellow for Tier 3, orange for Tier 4, and grey as NO ESCAT was found. Proportion of patients with the molecular alteration in the X-axes per glioma type.

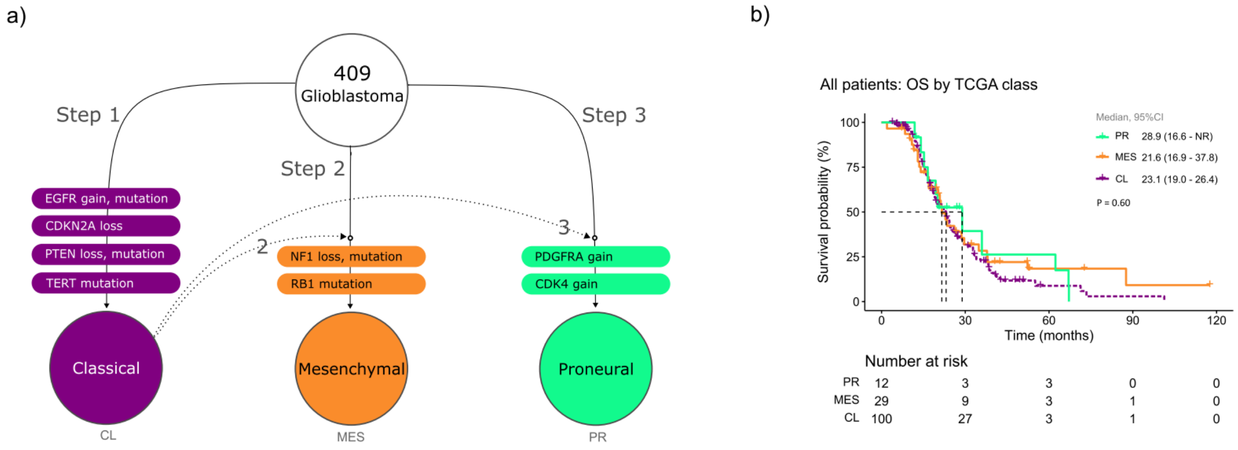

Supplementary Figure 3: Exploratory analysis of the adapted molecular classification with NGS information into classical, mesenchymal, and proneural (A), and the Kaplan Meier curves of the three groups (B).

**Legend:* The mutations that needed to be present to classify as classical were EGFR gain/mutation, CDKN2A/B loss, PTEN loss/mutation, and TERT mutation, to classify as mesenchymal were NF1 loss/mutation, and RB1 mutation, and to be classified as proneural PDGFRA gain or CDK4 gain. Note that IDH1 were excluded since now they are not considered GBM.*

Supplementary Tables

Supplementary Table 1: Patient demographic and tumor characteristics

	ALL Patients (N=541)	GBM (N=409)	Non-GBM (N=132)	Adjusted p-value*
**Age at dx in years, median (range)**	51.5 (3.14;83.6)	55.7 (3.14;83.6)	36.6 (16.9;70.8)	<0.001
Elderly (>60y)	154 (28.5%)	149 (36.4%)	5 (3.79%)	<0.001
Mid (>40;≤60y)	251 (46.4%)	209 (51.1%)	42 (31.8%)	<0.001
Young (≤40y)	136 (25.1%)	51 (12.5%)	85 (64.4%)	<0.001
**Gender**				
Male	330 (61.1%)	243 (59.6%)	87 (65.9%)	0.246
Female	210 (38.9%)	165 (40.4%)	45 (34.1%)	
**WHO 2021 Category**				<0.001
Astrocytoma – High Grade	29 (5.4%)	0 (0%)	29 (22%)	
Astrocytoma – Low Grade	76 (14%)	0 (0%)	76 (57.6%)	
GBM	409 (75.6%)	409 (100%)	0 (0%)	
Oligodendroglioma	27 (5%)	0 (0%)	27 (20.5%)	
**Verhaark Classification**				
Classical	201 (67.2%)	201 (67.2%)	0 (0%)	
Mesenchymal	61 (20.4%)	61 (20.4%)	0 (0%)	
Proneural	37 (12.4%)	37 (12.4%)	0 (0%)	
**TIER Group (ESCAT)**				<0.001
TIER 1-2	168 (31.1%)	36 (8.8%)	132 (100%)	
TIER 3-4	303 (56%)	303 (74.1%)	0 (0%)	
Unknown	30 (5.6%)	30 (7.3%)	0 (0%)	
Wild type	40 (7.4%)	40 (9.78%)	0 (0%)	

**IDH 1/2 Statues**				<0.001
Mutated	132 (24.4%)	0 (0%)	132 (100%)	
R132H	110 (83%)	0 (0%)	110 (83%)	
Non-canonical	22 (17%)	0 (0%)	22 (17%)	
Wild Type	409 (75.6%)	409 (100%)	0 (0%)	
**MGMT status**				0.061
Methylated	144 (44.6%)	107 (41.6%)	37 (56.1%)	
Not methylated	179 (55.4%)	150 (58.4%)	29 (43.9%)	
**Karnofsky status at dx**				
100	90 (17.8%)	52 (13.7%)	28 (29.9%)	
90	157 (31%)	119 (31.4%)	38 (29.9%)	
80	151 (29.8%)	116 (30.6%)	35 (27.6%)	
70	58 (11.3%)	48 (12.7%)	9 (7.1%)	
60	40 (7.9%)	34 (9%)	6 (4.7%)	
50	6 (1.2%)	5 (1.3%)	1 (0.8%)	
40	3 (0.6%)	3 (0.8%)	0 (0%)	
30	2 (0.4%)	2 (0.5%)	0 (0%)	
**ECOG status at dx**				0.058
0	146 (28.6%)	98 (25.6%)	48 (37.8%)	
1	283 (55.5%)	216 (56.4%)	67 (52.8%)	
2	71 (13.9%)	60 (15.7%)	11 (8.7%)	
3	8 (1.6%)	7 (1.8%)	1 (0.8%)	
4	2 (0.4%)	2 (0.5%)	0 (0%)	
**Survival Status**				
Alive	186 (34.4%)	111 (27.2%)	75 (56.8%)	
Cancer Death	293 (54.3%)	245 (60%)	48 (36.4%)	
Death by other causes	5 (0.9%)	5 (1.2%)	0 (0%)	
Lost of follow-up	56 (10.4%)	47 (11.5%)	9 (6.8%)	

**Matched therapy received**				0.045
Yes	47 (12.1%)	42 (14.3%)	5 (5.2%)	
No	343 (87.9%)	252 (85.7%)	91 (94.8%)	
**First procedure**				0.278
Biopsy	69 (13.1%)	48 (12.1%)	21 (16.3%)	
Surgery	458 (86.9%)	350 (87.9%)	108 (83.7%)	
**Surgery Response**				0.055
Complete resection	188 (36.6%)	148 (37.7%)	40 (33.3%)	
Incomplete resection	325 (63.4%)	245 (62.3%)	80 (66.7%)	
Number of treatment lines received	3 (1;10)	2 (1;9)	3 (1;10)	
Number of patients per Hospital				0.001
Vall d'Hebron	266 (49.2%)	221 (54%)	45 (34.1%)	
12 de Octubre	117 (21.6%)	78 (19%)	39 (29.6%)	
Clinic	48 (8.8%)	32 (7.8%)	16 (11.8%)	
Mar	33 (6.2%)	23 (5.7%)	10 (7.7%)	
Ramon y Cajal	29 (5.1%)	18 (4.5%)	10 (7.7%)	
ICO	28 (5.1%)	23 (5.7%)	5 (4%)	
Sant Pau	20 (3.7%)	14 (3.3%)	7 (5.3%)	

ECOG PS, Eastern Cooperative Oncology Group (ECOG) performance status. Dx= Diagnosis.

*All p-values are two-sided and were adjusted by the Benjamini and Hochberg (BH) method to account for the multiple comparisons issue.

Supplementary Table 2: Details of molecular alterations encountered for the entire cohort (n=541).

AKT1_E17K_MUT	0
AKT1_MUT	4
ATM_MUT	14
ATRX_MUT	80
BRAF_FUSION	8
BRAF_MUT	16
BRAF_V600E_MUT	7
CDK4_GAIN	43
CDK4_MUT	5
CDKN2A_LOSS	127
CDKN2B_LOSS	108
EGFR_AMP	128
EGFR_MUT	69
EGFRVIII_REARRANGEMENT	72
FGFR_1_3_AMP	3
FGFR_1_3_MUT	14
FGFR_REARRANGEMENT	13
H3K27M_MUT	11
IDH1_MUT	132
IDH2_MUT	10
JAK_CN	1
JAK_MUT	8
MDM2_CN	26
MDM2_MUT	3
MET_MUT	10
MYC_CN	3
MYC_MUT	1
NF1_CN	9
NF1_MUT	44
NTRK1_3_FUSION	10
PDGFRA_AMP	14
PI3K_MUT_PIK3CA_PTEN	149
POLE_CN	1
POLE_MUT	7
PTEN_LOSS	19
RB1_CN	4
RB1_MUT	22
ROS1_CN	0

ros1_mut	7
tert_mut	205
tp53_cn	5
tp53_mut	169

*Of note: not all panels had the same NGS coverage.

Supplementary Table 3: Descriptive table of all glioma patients separated by the modified molecular classification.

	Classical (N=201)	Mesenchymal (N=61)	Proneural (N=37)	Adjusted p-value*
**Age at dx in years, median (range)**	55.7 (20.9;80.5)	52 (12.8;77.4)	58.2 (30.2;78.5)	0.248
Elderly (>60y)	70 (34.8%)	22 (36.1%)	14 (37.8%)	
Mid (>40;≤60y)	114 (56.7%)	26 (42.6%)	21 (56.8%)	
Young (≤40y)	17 (8.5%)	13 (21.3%)	2 (5.4%)	
**Gender**				0.862
Male	125 (62.5%)	41 (67.2%)	24 (64.9%)	
Female	75 (37.5%)	20 (32.8%)	13 (35.1%)	
**WHO 2021 Category**				
Astrocytoma – High Grade				
Astrocytoma – Low Grade				
GBM	201 (67.2%)	61 (20.4%)	37 (12.4%)	
Oligodendroglioma				
**TIER Group (ESCAT)**				0.009
TIER 1-2	15 (7.5%)	5 (8.2%)	3 (8.1%)	
TIER 3-4	186 (92.5%)	51 (83.6%)	31 (83.8%)	
Unknown	0 (0%)	0 (0%)	0 (0%)	
Wild type	0 (0%)	5 (8.2%)	3 (8.1%)	
**IDH 1/2 Statues**				<0.001
Mutated	0 (0%)	0 (0%)	0 (0%)	
Wild Type	201 (100%)	61 (100%)	37 (100%)	

**MGMT status**				0.691
Methylated	58 (43%)	17 (56.7%)	10 (40%)	
Not methylated	77 (57%)	13 (43.3%)	15 (60%)	
**Karnofsky status at dx**				
100	20 (10.6%)	7 (12.5%)	6 (16.7%)	
90	68 (36.2%)	16 (28.6%)	14 (38.9%)	
80	62 (33%)	20 (35.7%)	6 (16.7%)	
70	18 (9.6%)	5 (8.9%)	7 (19.4%)	
60	13 (6.9%)	7 (12.5%)	2 (5.6%)	
50	4 (2.1%)	1 (1.8%)	0 (0%)	
40	2 (1.1%)	0 (0%)	1 (2.8%)	
30	1 (0.5%)	0 (0%)	0 (0%)	
**ECOG status at dx**				0.919
0	48 (25.4%)	15 (26.3%)	7 (19.4%)	
1	113 (59.8%)	33 (57.9%)	21 (58.3%)	
2	23 (12.2%)	8 (14%)	7 (19.4%)	
3	3 (1.6%)	1 (1.8%)	1 (2.8%)	
4	2 (1.1%)	0 (0%)	0 (0%)	
**Survival Status**				0.825
Alive	42 (20.9%)	18 (30%)	12 (32.4%)	
Cancer Death	125 (62.2%)	33 (55%)	21 (56.8%)	
Death by other causes	3 (1.5%)	1 (1.7%)	0 (0%)	
Lost of follow-up	31 (15.4%)	8 (13.3%)	4 (10.8%)	
**Matched therapy received**				0.825
Yes	22 (14.8%)	6 (12.5%)	5 (22.7%)	
No	127 (85.2%)	42 (87.5%)	17 (77.3%)	
**First procedure**				0.825
Biopsy	23 (11.6%)	7 (12.1%)	2 (5.4%)	

Surgery	175 (88.4%)	51 (87.9%)	35 (94.6%)	
**Surgery Response**				0.826
Complete resection	76 (38.8%)	25 (44.6%)	15 (40.5%)	
Incomplete resection	120 (61.2%)	31 (55.4%)	22 (59.5%)	
**Number of treatment lines received**	3 (1;7)	3 (1;7)	2 (1;6)	

ECOG PS, Eastern Cooperative Oncology Group (ECOG) performance status. Dx= Diagnosis.

*All p-values are two-sided and were adjusted by the Benjamini and Hochberg (BH) method to account for the multiple comparisons issue.
